# Targeting TGFBR1 with the Investigational Inhibitor TP-6379 Improves Hematopoiesis in Low-Risk MDS Ex Vivo

**DOI:** 10.3390/cells15181674

**Published:** 2026-09-16

**Authors:** Tiffany Razabdouski, Robert Dalton, Alexandra Calescibetta, Sean Christiansen, Grace Ward, Anthony C. Augello, Jungwon Moon, Seongseok Yun, Nicole Vincellette, Gabriela Wright, Annelise J. Glode, Dung-Tsa Chen, Jodi Kroeger, Jason M. Foulks, Steven L. Warner, Bin Duan, Bo Liu, Kenneth L. Wright, Erika A. Eksioglu

**Affiliations:** 1 Department of Immunology, Moffitt Cancer Center, Tampa, FL 33621, USA; tiffany.razabdouski@moffitt.org (T.R.); grace.ward@stjude.org (G.W.); annelise.glode@moffitt.org (A.J.G.); jodi.kroeger@moffitt.org (J.K.); ken.wright@moffitt.org (K.L.W.); 2 Cancer Biology PhD Program, University of South Florida and Moffitt Cancer Center, Tampa, FL 33612, USA; 3 Department of Malignant Hematology, Moffitt Cancer Center, Tampa, FL 33612, USA; anthony.augello@moffitt.org (A.C.A.); jungwon.moon@moffitt.org (J.M.); seongseok.yun@moffitt.org (S.Y.); nicole.vincelette@moffitt.org (N.V.); 4 Department of Bioinformatics and Biostatistics, Moffitt Cancer Center, Tampa, FL 33612, USA; dung-tsa.chen@moffitt.org; 5 Sumitomo Pharma America, Inc. (Formerly Tolero Pharmaceuticals), Marlborough, MA 01752, USA; 6 Mary & Dick Holland Regenerative Medicine Program, University of Nebraska Medical Center, Omaha, NE 68198, USA; bin.duan@unmc.edu (B.D.); bo.liu@unmc.edu (B.L.)

**Keywords:** TP-6379, ALK5/TGFBR1 inhibition, S100A9, hematopoiesis

## Abstract

**Highlights:**

**What are the main findings?**

- Specific ALK5 inhibition with TP-6379 has a significant effect on hematopoiesis in MDS primary specimens treated in vitro.
- TP-6379 reduced S100A9-mediated hematopoiesis dysregulation in vitro and in the S100A9-Tg mouse model in vivo.

**What are the implications of the main findings?**

- The use of TGFβ superfamily inhibition (i.e., Luspatercept) is approved for the treatment of anemia in low-/intermediate-risk MDS as a ligand trap.
- Targeting TGFβ with compounds for specific receptors may aid in preventing toxicities and enhance therapeutic efficacy against MDS pathology.

**Abstract:**

Myelodysplastic syndrome (MDS) is characterized by S100A9-mediated constitutive innate immune activation that leads to the inflammatory death of hematopoietic stem/progenitor cells (HSPCs). This suppressive microenvironment includes the secretion of TGFβ and is phenocopied by the S100A9-transgenic (Tg) mice. However, the role of the different ALK/TGFB receptors in hematopoiesis or in S100A9-mediated inflammation is not understood. Primary low-risk MDS bone marrow mononuclear cells (BM-MNCs) were analyzed in vitro for hematopoietic restoration by an ALK4/ALK5 inhibitor (Vactosertib), an ALK2 inhibitor (TP-0184/Itacnosertib) and an ALK5/TGFBR1-specific inhibitor (TP-6379). Specimens significantly responded to TP-6379 treatment restoring hematopoiesis, with a positive bias for specimens harboring spliceosomal mutations. We previously shown that S100A9 drives phenotypic pathology of MDS, and it has been known to induce and cooperate with TGFβ in this disease. Hence, we treated healthy BM-MNC with recombinant human (rh) S100A9 where TP-6379 rescued the S100A9-reduced hematopoiesis. The effect of TP-6379 was validated in vivo in S100A9-Tg mice. Similar results were obtained with K562 cells, with or without the knock-in (KI) mutation in SF3B1, with strong sensitivity to rhS100A9 that was overcome by TP-6379. Hence, directly targeting TGFBR1/ALK5 with TP-6379 restores healthy hematopoiesis in low-risk MDS preclinical models.

## 1. Introduction

Myelodysplastic syndrome (MDS) is a heterogeneous disease where the immunosuppressive microenvironment plays a key role in the ineffective hematopoiesis characteristic of the disease. We and others showed that the immunosuppressive effector S100A9 can directly kill hematopoietic stem and progenitor cells (HSPCs), through pyroptosis and induction of genotoxic stress, or indirectly by targeting HSPCs via recruitment of myeloid-derived suppressor cells (MDSCs) [1,2,3,4,5,6]. S100A9 binds to CD33, recruiting and enhancing the abundance of MDSCs in the bone marrow creating an immunosuppressive milieu through the secretion of meditators like TGFβ [4,7]. TGFβ is a suppressive cytokine with pleiotropic functionality, depending on the receptor sensing it, and has important effects on hematopoiesis and MDS pathogenesis [8]. Signaling of the TGFβ pathway, through the activation of SMAD2 in HSPC, has been shown to reduce the proliferation and differentiation of these cells [8]. This cytokine can in turn regulate and enhance the recruitment of MDSCs and promote tumorigenesis [9,10] and has been shown to be upregulated in low-risk MDS preclinical models [11]. MDSCs not only act through the increased secretion of TGFβ but through membrane-bound TGFβ1 which suppresses cytotoxic lymphocytes, NK cells and CD8^+^ T cells activity and IFNγ production [7,12]. However, the potential interaction between S100A9 and TGFβ, its therapeutic targetability, and its role in MDS remain poorly understood.

Currently, there are several therapeutic strategies in development focusing on different aspects of the immunosuppressive microenvironment in MDS including: targeting IL-1β (NCT04810611, NCT04239157) [13], preventing pyroptosis signaling (formation of ASC specs, caspase-1 activation or Gasdermin D, NCT03359460, NCT02553941) [13,14] and targeting the S100A9/CD33 axis to restore hematopoiesis in primary MDS ex vivo specimens [15,16]. Recently, targeting the TGFβ pathway with its wide array of suppressive functions has shown important therapeutic potential. In 2020 the FDA granted approval for Luspatercept, a recombinant fusion protein of the Activin receptor 2B used to trap excess endogenous TGFβ in the microenvironment improving erythropoiesis [8]. This TGFβ targeting strategy reduces transfusion burden in low-risk MDS patients with ring sideroblasts (MEDALIST (NCT02621070) clinical trial [17,18]). However, as a ligand trap Luspatercept is not highly specific which is compounded by TGFβ’s pleiotropic effects, which may be an important caveat of this targeting strategy as it is broad and not very selective. This can be noticed in the best responders to treatment that have lower baseline transfusion burden, are less likely to have received erythroid-stimulating agents (ESAs) and had to receive the maximum dose level to maintain benefit. Hence, refining which TGFβ pathway component, particularly its receptors, is specific to the therapeutic effects of these types of agents will be a key strategy to improve their efficacy.

In our current preclinical study, we used chemical inhibitors of three key ALK receptors including Vactosertib (ALK4/ALK5), TP-0184 (Itacnosertib, ALK2), and the investigational ALK5/TGFBR1-specific TP-6379 in bone marrow mononuclear cells (BM-MNCs) from either MDS patients or healthy donor BM-MNC treated with recombinant human (rh) S100A9 to assess restoration of hematopoiesis. We found that ALK5/TGFBR1-specific inhibition was consistently significant at increasing hematopoiesis, reducing MDSCs and reducing genomic instability in primary MDS specimens, and in vivo in the S100A9-Tg mouse model, suggesting ALK5/TGFBR1 inhibition as the important mediator of the TGFβ family in MDS.

## 2. Materials and Methods

### 2.1. Primary Specimens and Cell Lines

MDS patients provided informed consent on Institutional Review Board-approved protocols under Moffitt Cancer Center’s (MCC) Total Cancer Care (TCC) protocol [19]. Patients were consented through the TCC protocol (MCC#14690/Liberty IRB#104189) that serves as the Cancer Center’s general banking protocol. A waiver of HIPAA authorization was requested from the IRB for this biobanking protocol to permit the non-therapeutic research trial (NRTO) team and physicians to review patients’ medical charts to identify specimens that match the criteria for our protocol (MCC 20536). Patients undergoing bone marrow biopsy were recruited from the Malignant Hematology Clinic at the H. Lee Moffitt Cancer Center & Research Institute. Tissue specimens were collected and released for this study according to prior or predicted risk based on history and screening by MCC’s Tissue Core (RRID:SCR_012364). The pathological subtype of MDS was reported according to the World Health Organization (WHO) criteria and prognostic risk was assigned according to the International Prognostic Scoring System (IPSS). Unless indicated, samples used were from lower-risk (low, intermediate-1) MDS patients. BM aspirates were obtained from the Tissue Core post-isolation in heparinized tubes. Healthy (normal) heparinized BM aspirates were purchased from Lonza Walkersville or StemCell. Isolation of human BM-MNC was done using Ficoll-Hypaque Plus gradient centrifugation solution (Cat# 95038-168, Cytiva, Marlborough, MA, USA) using the SepMate-50 isolation tubes (StemCell, Cat# 85450) followed by removal of red blood cells with a Red Blood Cell Lysis Buffer (Cat# R7757, Sigma-Aldrich, Saint Louis, MO, USA). All experiments with primary MDS BM-MNCs were done fresh ex vivo; no frozen cells were used.

S100A9-Tg mice [3] were used for animal studies as we have defined them to be a model for inflammaging in MDS [4]. WT FVB/NJ mice were originally purchased from Jackson Laboratories and a colony was maintained in-house at Moffitt’s vivarium to ensure aged-matched animals for our experiments. All mouse studies were approved by the Institutional Animal Care and Use Committee at the University of South Florida. BM cells were isolated from tibias and femurs of experimental animals. Isolation of BM-MNCs was done using Ficoll-Hypaque Plus gradient centrifugation (Cat# 95038-168, Cytiva, Marlborough, MA, USA) followed by removal of red blood cells with a Red Blood Cell Lysis Buffer (Cat# R7757, Sigma-Aldrich, Saint Louis, MO, USA).

The WT and SF3B1 K700E KI mutation-bearing K562 cells (a common mutation in Splicing Factor 3b Subunit 1 (SF3B1) in MDS) were described previously [20] and had been used as a tool to understand the role of this mutation in hematopoiesis. They were obtained, courtesy of the Alan List lab previously at Moffitt Cancer Center. A synonymous change or mutation (c.2098A>G) was introduced using CRISPR/Cas9 genome engineering method. We maintained these cells in RPMI 1640 (Cat# 11875-119, Invitrogen, Carlsbad, CA, USA) supplemented with 10% fetal bovine serum (Cat# 100-500, GeminiBio, West Sacramento, CA, USA), 1% Pennicilin/Streptomycin (Cat# 15140-122, Invitrogen, Carlsbad, CA, USA), 1% Sodium Pyruvate (Cat# 25000CI, Corning, Corning, NY, USA), and 0.1% Mycozap Prophylactic (Cat# VZA-2032, Lonza Walkersville, Walkersville, MD, USA).

### 2.2. Reagents

Vactosertib was obtained (CAS no: 1352608-82-2, Cat# HY-19928, MedChem Express, Monmouth Junction, NJ, USA) and its specificity has been reported as ALK2 and ALK4 IC50 of 17.3 nM. TP-0184 and TP-6379 were provided by Sumitomo Pharma America. TP-0184 specificity has been reported as ALK2 with an IC50 of 5 nM and TP-6379 has a biochemical ALK5 IC50 of 1.07 nM [21,22,23,24]. TP-6379 has been shown to reduce pSMAD2 in Rhabdomyosarcoma cells with an IC50 of 108 nM [22].

### 2.3. Hematopoietic Colony-Forming Assay

One hundred thousand BM-MNCs were plated in duplicate onto SmartDish plates (Cat# 27301, StemCell Technologies, Vancouver, BC, Canada) in complete methylcellulose medium specific for human specimens (MethoCult Cat# H4434 Classic, StemCell Technologies, Vancouver, BC, Canada) or mice hematopoietic colonies (MethoCult GF Cat# M3434, Stemcell Technologies, Vancouver, BC, Canada) following the manufacturer’s recommendations. Dishes were incubated at 37 °C in 5% CO_2_ for 10–14 days, at which point colonies were counted on a StemVision microscope (Catalog # 22006, StemCell Technologies, Vancouver, BC, Canada) using the CFU counting software of the instrument. The software colony count was then validated by manual recounting. The specimens used in colony assays are tabulated in Supplementary Table S1.

### 2.4. Flow Cytometry

Labeling was carried out after washing with 1X PBS (Cat# 14190-250, Invitrogen, Carlsbad, CA, USA). Cells were harvested, washed twice in 1X PBS, and stained with live/dead fluorescent reactive dyes according to the manufacturer’s protocol for PBS in the absence of protein to avoid non-specific staining (Blue Cat# L34961, Aqua Cat# L34965, Violet Cat# L34963, Near IR Cat# L34992, all from Invitrogen, Carlsbad, CA, USA). After washing to remove the live/dead reagent, non-specific binding was blocked by incubating cells in flow-cytometry-staining buffer containing 1X PBS supplemented with 0.5% bovine serum albumin (BSA, Cat# 0332-500G, VWR life science, Radnor, PA, USA), 0.1% human male AB serum (Cat# 515-H1, Access Cell Culture, Vista, CA, USA) and 0.1% mouse serum (Cat# 31881, Thermo Fisher Scientific, Waltham, MA, USA). To block Fc receptors, we used the TrueStain FcX block (Cat# 422302, Biolegend, San Diego, CA, USA) at room temperature for 15 min. Cells were subsequently stained with specific conjugated antibodies and diluted in the same buffer for 1 h at room temperature. Following washing, cells were fixed with BD Cytofix/Cytoperm buffer (Cat# BDB554722, BD Biosciences, Franklin Lakes, NJ, USA) and washed with perm/wash buffer (Cat# BDB554723, BD Biosciences, Franklin Lakes, NJ, USA) for experiments requiring intracellular staining analysis. Sample acquisition was carried out using a BD LSRII flow cytometer or an Aurora cytometer (Cytek^®^ Aurora, Cytek^®^ Biosciences, Fremont, CA, USA), located at Moffitt’s Flow Cytometry Core equipped with the FACSDiva software version 9.3 (BD Biosciences, Franklin Lakes, NJ, USA). Data was analyzed using FlowJo software (FlowJo, LLC., Ashland, OR, USA).

The following antibodies were used in this study for flow cytometry.

#### 2.4.1. Murine Panel

Antibodies from BD Biosciences (Franklin Lakes, NJ, USA) include: rat anti-mouse Ly-6C and Ly-6G (Gr-1) FITC clone RB6-8C5 (Cat# 553127), and rat anti-mouse Ly6-A/E (Sca1) BUV395 clone E13-161.7 (Caat#744328). Antibodies from Biolegend (San Diego, CA, USA) include: anti-mouse/human CD11b PE/Cyanine7 clone M1/70 (Cat# 101216), anti-mouse CD117 (c-kit) PerCP/Cyanine5.5 clone 2B8 (Cat# 105824), and anti-mouse Lineage Cocktail Brilliant Violet 421 clones: 17A2; RB6-8C5; RA3-6B2; Ter-119; M1/70 (Cat# 133311). Antibodies for the in vivo mobilization assay include: mouse anti-human CD45 BUV805 clone HI30 (Cat# 612891, BD Biosciences, Franklin Lakes, NJ, USA), mouse anti-human CD11b AlexaFluor 488 (Cat# 557701, BD Biosciences, Franklin Lakes, NJ, USA), and anti-human CD3 eFluor450 clone OKT3 (Cat# 48-0037-42, Invitrogen, Carlsbad, CA, USA).

#### 2.4.2. Human Panel

Antibodies from BD Biosciences (Franklin Lakes, NJ, USA) include: mouse anti-human CD45 APC-H7 Clone 2D1 (Cat# 560274), mouse anti-human CD33 BB515 clone WM53 (Cat# 564588), mouse anti-human CD14 BUV737 clone M5E2 (Cat# 612764), mouse anti-human HLA-DR PE-Cy7 clone G46-6 (Cat# 560651), rat anti-human CD11b APC-R700 clone M1/70 (Cat# 564985), mouse anti-human CD16 BUV395 (Cat# 563784), mouse anti-human NCAM-1 (CD56, Cat# 563238), mouse anti-human CD3 BUV805 clone SK7 (also known as Leu-4, Cat# 612894), mouse anti-human CD34 APC clone 581 (Cat# 560940), mouse anti-human CD4 BV711 clone SK3 (also known as Leu3a, Cat# 563033), mouse anti-human CD8 BUV563 clone RPA-T8 (Cat# 612915), mouse anti-human CD19 BB700 clone SJ25C1 (also known as SJ25-C1, Cat# 566397), and mouse anti-human CD25 BV480 clone M-A251 (Cat# 566160). Antibodies from Biolegend (San Diego, CA, USA) include: mouse anti-human CD15 BV421 clone W6D3 (Cat#323039), mouse anti-human CD57 BV785 clone QA17A04 (Cat# 393329), and mouse anti-human CD127 BV650 clone A019D5 (Cat# 351325).

#### 2.4.3. Surface Expression and Intracellular Signaling Analysis

The involved antibodies include: anti-human TGFBR1 FITC (Cat# LS-C499925, Lifespan Biosciences, Lynnwood, DC, USA), rabbit anti-human SMAD2/3 PE clone D7G7 (Cat# 72255S, Cell Signaling Technology, Danvers, MA, USA), rabbit anti-human p-SMAD (S465/S467) Alexa 647 (Cat#68559S, Cell Signaling Technology, Danvers, MA, USA), anti-human MRP-14 (S100A9) FITC clone MRP 1H9 (Cat#350704, Biolegend, San Diego, CA, USA), mouse anti-human TLR-4 (CD284) PE clone HTA125 (Cat# 12-9917-73, ebioscience, San Diego, CA, USA), mouse anti-human CD33 BV786 clone HIM3-4 (Cat#744355, BD Biosciences, Franklin Lakes, NJ, USA), anti-human RAGE unconjugated clone DD/A11 or mAbA11 (Cat# MAB5328, Millipore) secondary goat anti-mouse IgG AlxaFluor 647 (Cat# A21236, Invitrogen, Carlsbad, CA, USA), rabbit anti-human SERPIN E1/PAI-1 AlexaFluor 405 (Cat# NBP1-19773AF405, Novus, St. Louis, MO, USA), anti-human Lineage Cocktail (CD3/14/16/19/20/56) Pacific Blue clones (UCHT1; HCD14; 3G8; HIB19; 2H7; HCD56 (Cat#348805, Biolegend, San Diego, CA, USA)), and anti-human CD34 PerCP-Cy5.5 clone 8G12 (Cat# 3447213, BD Biosciences, Franklin Lakes, NJ, USA).

For cell cycle analysis, BM-MNCs from either MDS or healthy donors were treated with TP-6379 in the presence or absence of rhS100A9. For each experimental condition, 1 million cells were fixed in cold 70% ethanol for 1 h followed by washing with 1X PBS twice. Pelleted cells were resuspended in a 1 mL mixture containing 5 µg/mL Ribonuclease A (Cat# R6513, Sigma-Aldrich, Burlington, MA, USA) and 10 µg/mL DAPI (Cat# FP1490, PerkinElmer, Shelton, CT, USA) in 1X PBS. The cells were stained for 20 min at room temperature in the dark, then washed twice with 1X PBS and resuspended in 500 µL of 1X PBS for analysis by flow cytometry (BD LSRII, BD Biosciences, Franklin Lakes, NJ, USA.

### 2.5. RNA Isolation, cDNA Synthesis, and Real-Time PCR

Cells were harvested and RNA was isolated using the RNeasy Mini Kit (Cat# 74104, Qiagen, Germantown, MI, USA) following the manufacturer’s protocol. RNA concentration was measured using a Nanodrop Spectrophotometer (NanoDrop One, Thermo Fisher Scientific, Waltham, MA, USA) and 1 µg of RNA was used for reverse transcription (RT) in a 20 µL volume (QSCRIPT CDNA Supermix, Cat# 95048-100, Quanta Bio, Beverly, MA, USA). A “no RT” control was used for all experiments to confirm the absence of DNA contamination. For quantitative real-time RT-PCR (Perfecta SYBR Fastmix, Cat# 95072, Quanta Bio, Beverly, MA, USA), 1 uL of cDNA was used for each reaction. All samples were done in triplicate, and reactions were conducted in a 96-well spectrofluorometric thermal cycler (CFX96 real-time detection system, Bio-Rad, Hercules, CA, USA). The PCR conditions were as follows: 95 °C for 3 min followed by 40 cycles of 95 °C, 15 s; 60–62 °C (primer dependent), 30 s; and 95 °C, 1 min. A melting curve was added to every run for quality assessment starting at 55 °C and ramping by 0.5 °C for 80 repeats. Relative fold changes in expression were determined by using the comparative cycle threshold method (2−ΔΔCT). The following primers were used: *SERPINE1* forward 5′-CTCATCAGCCACTGGAAAGGCA-3′; *SERPINE1* Reverse-5′-GACTCGTGAAGTCAGCCTGAAAC-3′; *TXNIP* forward-5′-CAGCAGTGCAAACAGACTTCGG-3′; *TXNIP* reverse-5′-CTGAGGAAGCTCAAAGCCGAAC-3′; *18S* forward-5′-CGGCTACCACATCCAAGGAAGG-3′; and *18S* reverse-5′-CCCGCTCCCAAGATCCAACTAC-3′. The housekeeping gene *18S* served as the internal PCR control.

### 2.6. Confocal Immunofluorescence Microscopy

After appropriate culturing conditions for each experiment, cells were collected on slides in a Cytospin by centrifugation for 3 min at 500 rpm and fixed with BD Cytofix/Cytoperm buffer (Cat# BDB554722, BD Biosciences, Franklin Lakes, NJ, USA) for 15 min at room temperature followed by 3 washes with 1X PBS (pH of 7.4) and freezing at −20 °C until use. When ready, slides were allowed to defrost, rehydrated with three 5 min washes with 1X PBS followed by blocking with 2% bovine serum albumin (BSA, cat# 0332-500G, VWR life science, Radnor, PA, USA) in PBS for 30 min at room temperature. Primary antibodies used for confocal microscopy analysis were anti-үH2AX (Cat# NB100-384, Novus Biologicals Inc, Centennial, CO, USA), anti-RNA:DNA hybrids (Clone S9.6, Cat# ENH001, Kerafast, Boston, MA, USA) and anti-PAI-1 antibody (cat# ab66705, Abcam, Boston, MA, USA). Primary antibodies were diluted in blocking buffer used to incubate the slides overnight at 4 °C. The next day the slides were washed three times with 1X PBS after which the appropriate secondary antibodies were applied: AlexaFluor 488 goat anti-mouse IgG (Cat# A11001, Invitrogen, Carlsbad, CA, USA), CF647 goat anti-rabbit IgG (cat#20282, Biotium, Freemont, CA, USA), and donkey anti-rabbit AleaFluor 488 (Cat# A21206, Invitrogen, Carlsbad, CA, USA). These antibodies were diluted in blocking buffer and incubated on the slides for 1 h in the dark at room temperature. Slides were then washed and mounted with ProLong Gold Antifade Reagent with DAPI (Cat# P36931, Invitrogen, Carlsbad, CA, USA) and covered with a coverslip. Fluorescence imaging was measured with a Leica TCS SP5 AOBS Laser Scanning Confocal microscope (Leica Microsystems GmbH, Wetzlar, Germany) at the Microscopy Core Facility of the Moffitt Cancer Center where samples were excited with 405, 488, and 638 diode lasers and emissions were tuned to appropriate settings for each dye. Images were captured through a 40X/1.4NA objective lens using two PMT detectors (DAPI and Alexa 488) and one HyD detector (Alexa 647). All system settings remained consistent for all samples within each experiment. Images were viewed and exported in TIFF format with LAS X software version 3.1.5 (Leica Microsystems GmbH, Wetzlar, Germany).

### 2.7. Statistical Analysis

Statistical analyses were performed using GraphPad Prism version 9.4.1 (GraphPad Software, San Diego, CA, USA). Differences in colony-forming capacity were evaluated using two-way ANOVA followed by Tukey’s multiple comparison test. For murine and human immunophenotypic flow cytometry analyses, potential outliers were identified using the ROUT (Robust Regression and Outlier Removal) method (Q = 5%). Data normality was assessed using the Shapiro–Wilk test. Normally distributed data were analyzed using repeated-measures one-way ANOVA, whereas non-normally distributed data were analyzed using the Kruskal–Wallis test. Comparisons between two groups were performed using two-tailed Student’s *t* tests, with paired or unpaired tests applied as appropriate based on the experimental design if the experiments were not matched/repeated measures. All statistical tests were two-sided, and *p*-values < 0.05 were considered statistically significant. Data were presented as mean ± SEM unless otherwise indicated.

## 3. Results

### 3.1. Specific Inhibition of ALK5/TGFBRI Restores Hematopoietic Colony-Forming Capacity in Primary Low-Risk MDS Specimens

**Figure 1:**
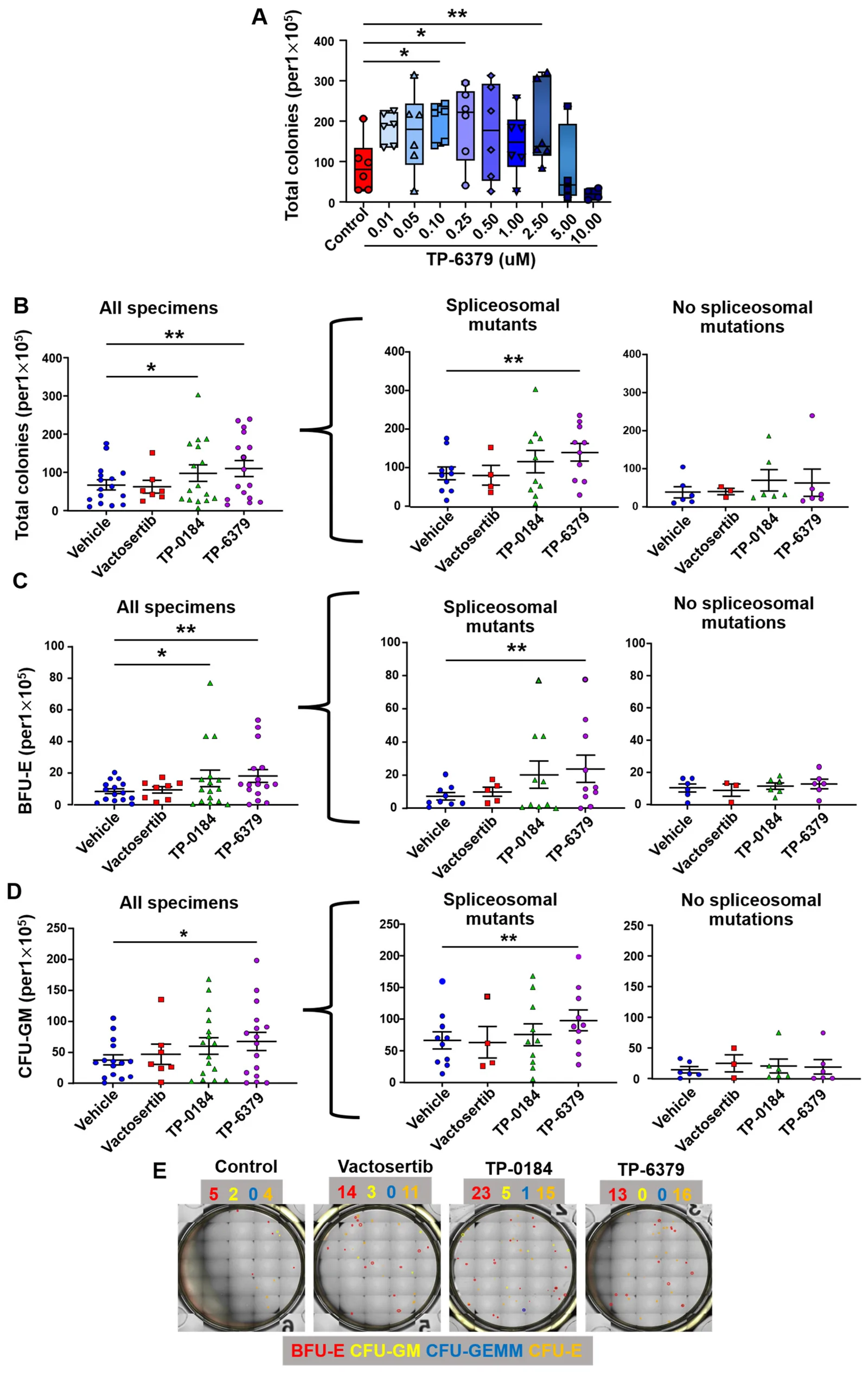
**Treatment of cells with TP-6379 observed to increase hematopoietic colony-forming capacity in primary MDS explants.** (**A**) Total colonies of primary human low-risk MDS BM-MNCs treated ex vivo for 48 h in the presence of increasing doses (0.01 to 10 uM) of TP-6379 diluted in DMSO (vehicle). Primary human low-risk MDS BM-MNCs (Supplementary Table S1) were treated for 48 h in the presence of 0.1μM of Vactosertib, TP-0184 or TP-6379 diluted in DMSO (vehicle) prior to assessing colony-forming capacity. (**B**) Total colonies, (**C**) BFU-E and (**D**) CFU-GM colonies of patient specimens (all, left panel) or the same specimens separated into spliceosomal (middle panels) and non-spliceosomal mutants (right panels). In all figures, error bars represent the standard error of the mean (SEM) and the middle line is the average of data points shown. Data was analyzed by ANOVA with multiple comparison analysis. *p*-values are shown as asterisk: * *p* ≤ 0.05 and ** *p* ≤ 0.01 as summarized in Graphpad Prism. (**E**) Representative colonies from a specimen from a 61-year-old female with single lineage dysplasia and no mutation that was resistant to Procrit.

TGFβ and its receptors have been shown to play a key role in MDS but most of the therapeutic strategies used have been broad. To understand which ALK receptor plays a prominent role in hematopoiesis of low-risk MDS we obtained primary BM-MNCs through the Total Cancer Care protocol at the Moffitt Cancer Center [19] to study the effect of different ALK inhibitors on hematopoiesis in vitro. While the effective dose for inhibition of pSMAD2 has been established for TP-0184 (IC50 of 17.3 nM) and TP-6379 (IC50 of 1.07 nM [21,22,23,24]) with optimal dose of 108 nM for the latter, the effective dose for ex vivo hematopoietic colony-forming ability of these compounds could be different. Hence, we first tested MDS primary specimen BM-MNCs in vitro in colony-forming assays with a dose range of 0.01µM to 10µM. The minimal dose at which TP-6379 significantly increased colonies was 0.1 µM, although these same doses with TP-0184 did not (Figure 1A and Supplementary Figure S1A).

Hence, 0.1µM was chosen as the optimal dose for inducing colonies and cultured in vitro with vehicle (DMSO) or a 0.1µM concentration of either Vactosertib (an ALK4/ALK5 inhibitor), TP-0184 (an investigational ALK2 inhibitor) or TP-6379 (an investigational ALK5/TGFBRI-specific inhibitor) for 48 h prior to plating in methylcellulose. A total of 16 MDS BM-MNC specimens were tested in vitro for most compounds although TP-0184 and TP-6379 were prioritized over Vactosertib since some of the specimens did not have enough cells for all assessments. The characteristics of the specimens used for in vitro colony-forming assays are shown in Supplementary Table S1. The original risk assessment was based on medical history which was then validated after bone marrow pathological assessment confirming low–intermediate risk status. At the selected dose of 0.1 µM we did not see changes in viability of BM-MNCs after treatment for 72 h (Supplementary Figure S1B). However, it is important to note that TP-0184 doses from 2.5 µM and above reduced colony formation, while TP-6379 only reduced colony formation at a dose of 10 µM but it was not significantly different from control (Figure 1A and Supplementary Figure S1A).

Total hematopoietic colonies and BFU-E (blood forming units-erythroid) colonies were significantly increased after treatment with TP-0184 or TP-6379 but not with Vactosertib (Figure 1B,C). However, TP-6379 was the only compound able to significantly increase CFU-GM (colony forming unit-GM) colonies (Figure 1D). When analyzing we noticed that MDS specimens with spliceosomal mutations (i.e., SF3B1, SRSF2; stratified as described previously [25]) responded to TP-6379 specifically while there was no significant increase when analyzing non-spliceosomal mutant specimens (same specimens as left panel separated in middle and right panels for visualization in Figure 1B–D). However, it is important to note that TP-0184, while not significant, also had a trend upward in the restoration of hematopoietic colonies at that dose. This may be due to the more rapid metabolism of Vactosertib which has been shown clinically to not accumulate leading to a need for potential repeated doses to achieve its effect [26]. We also observed an increase in colony-forming capacity after treatment of BM-MNCs in vitro from our specimen cohort that were refractory to Procrit (Epoetin alfa) or other therapeutic options although we did not have enough specimens to show statistical analysis of this last observation (representative specimen in Figure 1E and Supplementary Figure S1D). We separated specimens that were from ring-sideroblast-presenting patients (*n* = 3) or had been resistant to Procrit (*n* = 4) or Luspatercept (*n* = 2) and while not enough for statistical inference it shows that all these specimens increased colonies after treatment with TP-6379 (Supplementary Figure S1C–E). It is important to emphasize that we waited for over a year prior to submission to wait for extra specimens in these categories but were ultimately unsuccessful. Therefore, future studies could look at the potential of specific ALK5 inhibition in these rare populations.

### 3.2. Specific Inhibition of ALK5/TGFBR1 Rescues S100A9-Induced Hematopoietic Colony Deficiency in Healthy BM-MNCs

**Figure 2:**
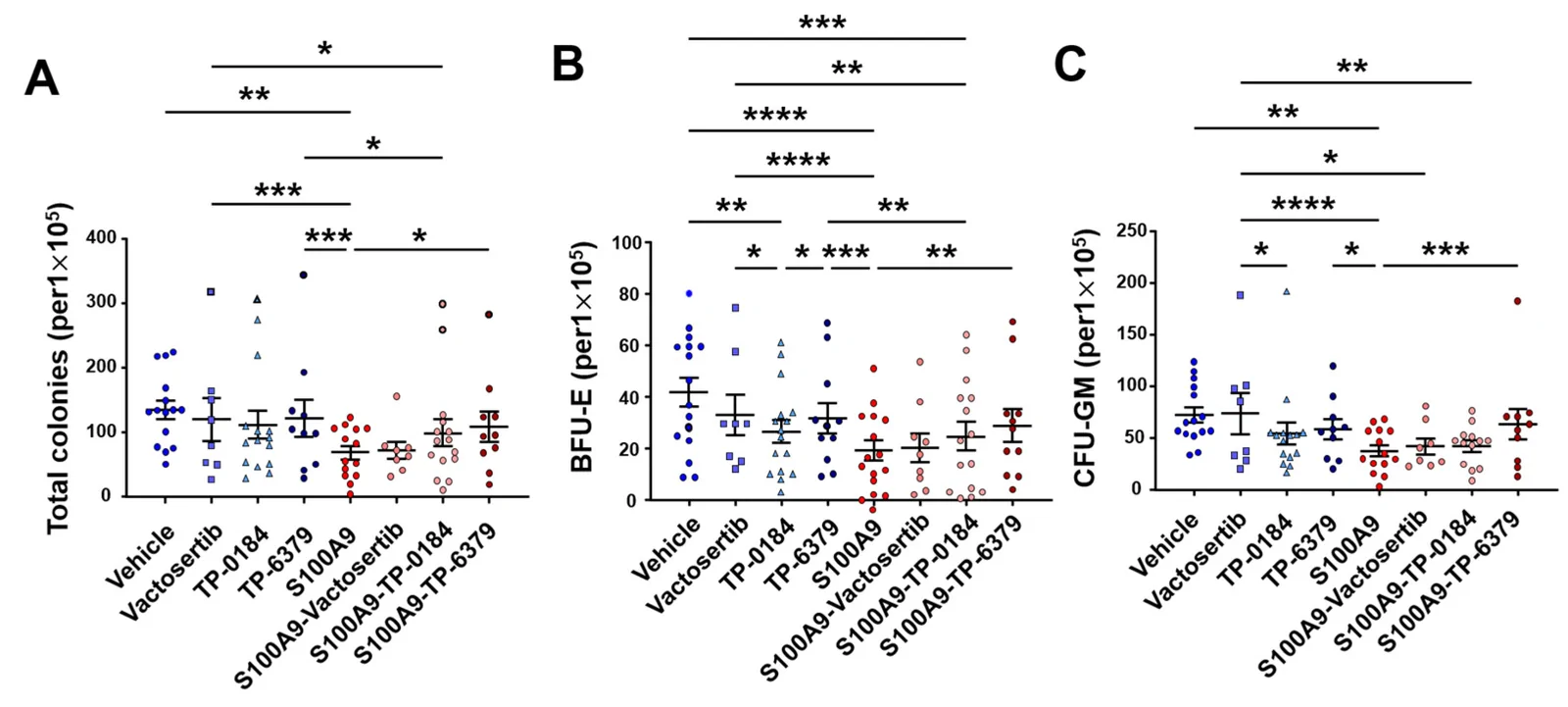
**Treatment of cells with TP-6379 shown to restore hematopoietic colony-forming capacity reduced by rhS100A9.** Primary human healthy BM-MNCs were treated for 48 h with 0.1 μM of Vactosertib, TP-0184 or TP-6379 diluted in DMSO (vehicle) in the presence (red markers) or absence (blue markers) of 10 µg/mL of rhS100A9. Hematopoietic colony-forming capacity was then assessed and analyzed as (**A**) total colonies, (**B**) BFU-E and (**C**) CFU-GM colonies. Data was analyzed by ANOVA with multiple comparison analysis. *p*-values are shown as asterisk: * *p* ≤ 0.05, ** *p* ≤ 0.01, *** *p* ≤ 0.001, and **** *p* ≤ 0.0001 as summarized in Graphpad Prism.

We have previously demonstrated the role that human S100A9 has in the immune pathogenesis of MDS, and in the maintenance of a suppressive microenvironment through the secretion of mediators such as TGFβ [4,5]. Moreover, others have demonstrated the role of this protein in bone marrow damage linked to disease pathogenesis [1,6] and we have shown that it is a targetable marker for therapeutic development [5]. While the link between S100A9 and TGFβ in TGFβ-targeted therapies is not clearly defined, it is known that S100A9 and TGFβ are in a feedback loop [27] and synergize to recruit myeloid cells to the tumor microenvironment [28]. S100A9-induced pyroptosis produces IL-1β which cooperates with TGFβ to enhance immunosuppressive signaling [5,29]. However, it is not known if targeting ALK5/TGFBR1 would allow the disruption of S100A9’s feed-forward mechanism. To test this hypothesis, we cultured healthy human BM-MNCs in the presence or absence of recombinant human (rh) S100A9 while treating with the three TGFβ pathway inhibitors as performed for primary MDS specimens. Treatment with these compounds in the absence of rhS100A9 did not significantly alter total colony formation suggesting that the compounds themselves do not modify healthy hematopoiesis (Figure 2A, blue markers). However, TP-0184 did decrease the proportion of BFU-E (Figure 2B, blue markers) and CFU-GM (Figure 2C, blue markers). As expected, all colonies were significantly reduced in the presence of rhS100A9 which was significantly and consistently restored in all colony types by TP-6379 only (Figure 2A–C, red markers). This suggests that ALK5/TGFBR1 inhibition specifically restored S100A9-reduced colony-forming capacity.

### 3.3. In Vivo Inhibition of ALK5/TGFBR1 Improves Anemia in the S100A9-Tg Mice

An important preclinical validation on the role of S100A9 is the use of S100A9-transgenic (Tg) mice (overexpressing murine S100A9 in hematopoietic cells). We developed these mice who have become an MDS model through its ability to induce the age-dependent inflammatory milieu that recapitulates characteristic features of MDS including histological analysis where it was observed: development of severe pancytopenia, hypercellularity (95% cellularity), tri-lineage cytological dysplasia, dysplastic megakaryocyte morphology, preponderance of mononuclear or hypolobated forms, erythroid precursors displaying megaloblastoid maturation with abnormal hemoglobination and occasional binucleation, nuclear budding and bizarre mitotic forms and increased percentage of myeloid lineage cells identified by myeloperoxidase staining compared to age-matched wild type controls [4]. Moreover, we and others have used these S100A9 overexpressing mice to study MDS inflammatory processes and therapeutic preclinical development in the past making it an ideal model to understand the potential of TP-6379 [1,2,5,6,15].

**Figure 3:**
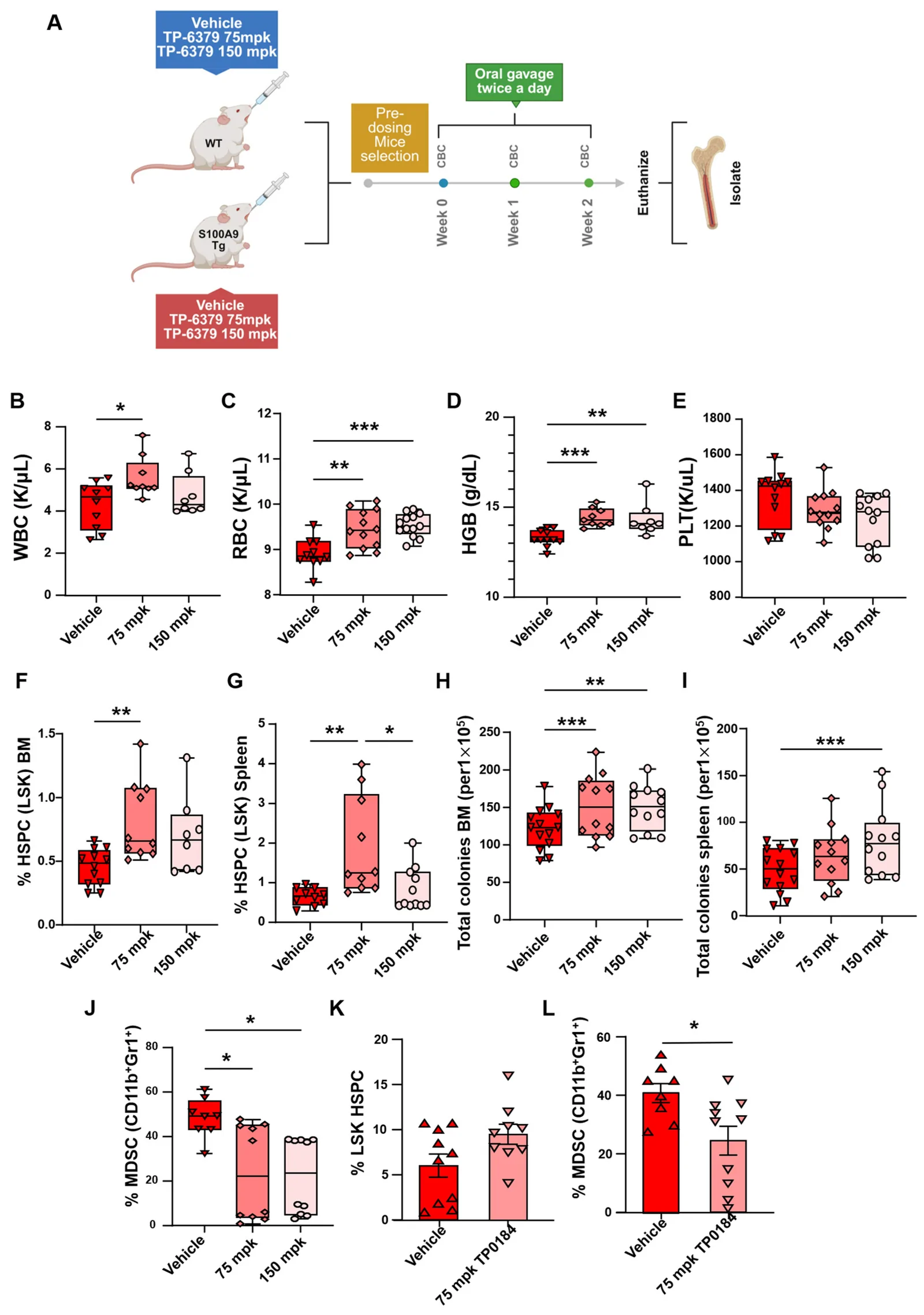
**TP-6379 shown to improve hematopoiesis in S100A9-Tg mice.** S100A9-Tg mice aged 9–12 months old (*n* = 13) were treated with TP-6379 at either 75 mg per kilo (mpk) or 150 mpk twice a day via oral gavage for two weeks as schematized in (**A**) (created in BioRender. Eksioglu, E. (2026) https://BioRender.com/46kyp0p). Complete blood counts were performed prior to study end (two weeks) where the levels of (**B**) white blood cells (WBCs), (**C**) red blood cells (RBCs), (**D**) hemoglobin (Hgb) and (**E**) platelets (PLTs) were measured. At study end (two weeks), bone marrow and spleens were collected and MNCs analyzed by flow cytometry (gating strategy in Supplementary Figure S2C). Percentages of HSPCs in the bone marrow (BM) (**F**) and spleen (SP) (**G**) were analyzed to confirm hematopoietic improvement and validated by colony-forming assays of BM (**H**) and spleen (**I**) from a total of 1 × 10^5^ mononuclear cells after 14 days in methocult media with appropriate murine growth factors. We also measured the percentages of the Gr1^+^CD11b^+^ MDSC compartments from both doses in the bone marrow (**J**). The same in vivo experiment was carried out with TP-0184 at 75 mpk for two weeks at which point the percentages of (**K**) HSPCs and (**L**) MDSCs in the total live cells were measured. Data was analyzed by ANOVA with multiple comparison analysis. *p*-values are shown as asterisk: * *p* ≤ 0.05, ** *p* ≤ 0.01 and *** *p* ≤ 0.001 as summarized in Graphpad Prism.

To validate our observation on the link between S100A9, TGFBR1 and hematopoiesis we treated aged S100A9-Tg mice [3,4], or aged-matched wild type (WT) counterparts, with either vehicle or 75 and 150 milligrams per kilo (mpk) of TP-6379 via oral gavage twice a day 10 h apart for two weeks (scheme in Figure 3A). Complete blood counts at week 2 prior to euthanasia demonstrated a significant improvement in the S100A9-induced anemia demonstrated by a significant increase in white blood cells (WBCs) at 75 mpk (Figure 3B), red blood cells (RBCs) (Figure 3C), and hemoglobin (HGB) (Figure 3D), but not platelets (PLTs) (Figure 3E), after TP-6379. Treatment of aged-matched WT mice did not induce any changes in WBC counts (Supplementary Figure S2A) or RBCs (Supplementary Figure S2B) at the two-week treatment mark compared to vehicle control mice, as expected as these are the non-anemia levels. After euthanasic BM and spleens were harvested and MNCs analyzed by flow cytometry for changes in HSPCs (defined as lineage^−^cKIT^+^Sca1^+^ cells, Supplementary Figure S2C), both BM (Figure 3F) and spleen (Figure 3G) HSPCs significantly increased at the 75 mpk dose, with a concomitant increase in colony-forming capacity in both hematopoietic organs after treatment (Figure 3H,I). Noticeably the 150 mpk dose did not significantly increase HSPCs in BM nor spleen but did have a significant effect on the colony-forming capacity of hematopoietic progenitors in both BM and spleen. Neither dose of TP-6378 affected the HSPC proportions in WT mice suggesting that the ALK5/TGFBR1 inhibition with TP-6379 does not affect healthy HSPCs after treatment for two weeks (Supplementary Figure S2D,E).

We have shown that MDSCs are induced by S100A9 and direct contributors in the microenvironment leading to death of HSPCs [4]. Therefore, we also analyzed the changes in the proportion of MDSCs by flow cytometry (defined as CD11b^+^Gr1^+^ cells, Supplementary Figure S2C). Both 75 mpk and 150 mpk significantly decreased these populations after treatment with TP-6379 (Figure 3I) while it did not significantly affect these cells in WT mice after treatment (Supplementary Figure S2F,G). To confirm that TP-6379 is more active in S100A9-induced pathology we repeated the experiment at the 75 mpk dose with TP-0184 and while we only observed an increased trend for HSPCs (Figure 3J) we saw a significant reduction in MDSCs (Figure 3K), akin to our observations in human BM-MNCs treated with rhS100A9 and TP-0184. This confirms that ALK5/TGFBR1 inhibition is also able to improve S100A9-mediated anemia in S100A9-Tg mice after treatment and that TP-6379-mediated ALK5 inhibition is more active than ALK2 inhibition with TP-0184 in reducing the pathological effects of S100A9 in the microenvironment and restoring hematopoiesis.

### 3.4. TP-6379 Reduces Active SMAD2 in HSPCs and MDSCs, Reduces Genomic Instability and Increases T Cell Populations in MDS

**Figure 4:**
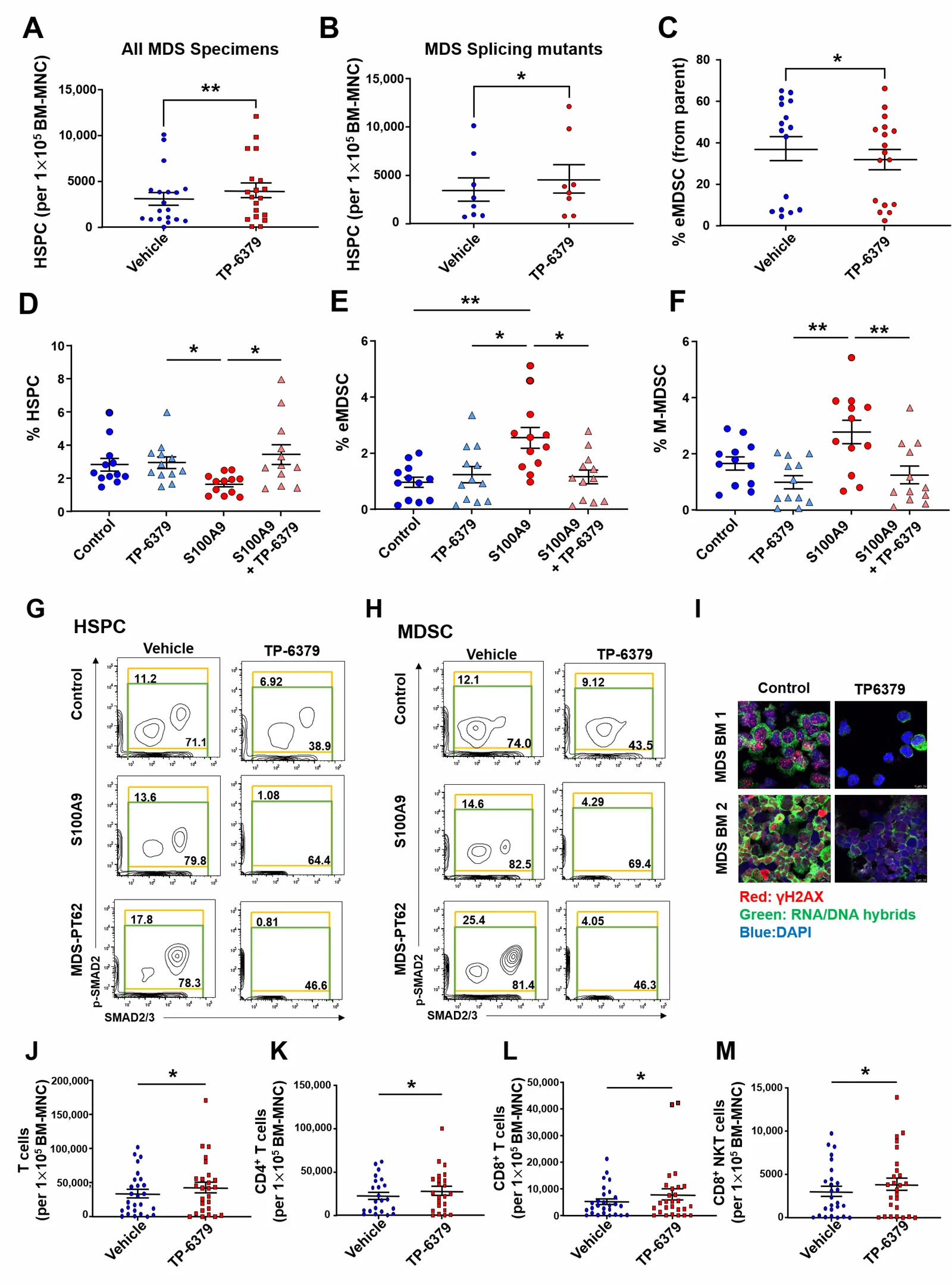
**MDSCs decrease, and lymphocytes increase after TP-6379 treatment of primary BM-MNCs.** Primary BM-MNCs were treated for 48 h with 0.1 μM of TP-6379 and immunophenotyped by flow cytometry (gating strategy is in Supplementary Figure S3 and specimens characteristics are in Supplementary Table S2). (**A**) Percentage of HSPCs in total live cells of low-risk MDS specimens or (**B**) the spliceosomal mutants only (*n* = 10). (**C**) Percentage of eMDSCs in low-risk specimens. Percentages of HSPCs (**D**), eMDSCs (**E**) and mMDSCs (**F**) in healthy MNCs treated with TP-6379 in the presence or absence of rhS100A9 measured by flow cytometry. Phosphorylation status of SMAD2 (yellow gate) and total positive SMAD2/3 cells (green gates) were analyzed for (**G**) HSPCs and (**H**) MDSCs from either a healthy donor treated with S100A9 or an MDS patient specimen (PT-62) after treatment with TP-6379 (representative specimens from 3 separate trials). (**I**) Reduction in genomic instability, measured by γH2AX by immunofluorescence staining (red), and formation of RNA:DNA hybrids (green) after treatment with TP-6379 (two representative specimens shown). Percentages of (**J**) T cells, and subpopulations (**K**) CD4^+^ T cells and (**L**) CD8^+^ T cells, as well as (**M**) CD8^+^ NKT cells in total live cells gated as in the immunophenotyping panel described in (**A**). Data was analyzed by ANOVA with multiple comparison analysis. *p*-values are shown as asterisk: * *p* ≤ 0.05 and ** *p* ≤ 0.01 as summarized in Graphpad Prism.

To fully understand how ALK5/TGFBR1 inhibition affects the immune microenvironment in MDS we developed a flow cytometry panel to profile HSPCs, subtypes of MDSCs, and lymphocytes (gating strategy, Supplementary Figure S3A; specimen characteristics in Supplementary Table S2). Just as observed with colony-forming capacity, the HSPC population in human MDS BM significantly increased in the mononuclear population after treatment with TP-6379, including the spliceosomal mutant specimens (Figure 4A,B). In the case of MDSCs, the most immature subtype of MDSCs, eMDSC, was significantly downregulated in MDS BM after treatment (Figure 4C). In S100A9-stimulated healthy BM-MNCs, HSPCs were restored after TP-6379 treatment while it reduced not only eMDSCs but also M-MDSCs (Figure 4D–F) suggesting TP-6379 affects the S100A9 feed-forward mechanism. Both HSPCs and MDSCs had reduced levels of phosphorylated SMAD2 cells after treatment with TP-6379 (gates in yellow, Figure 4G,H). Treatment of healthy BM-MNCs with rhS100A9 induced this activation as expected in both, and which was abrogated by TP-6379 treatment. The level of total SMAD2/3 was also strongly decreased after treatment but only in rhS100A9-treated or MDS BM-MNCs (gates in green, Figure 4G,H). This demonstrates that TP-6379 is actively inhibiting the TGFBR1 pathway in HSPCs and MDSCs.

MDS is a disease with a capacity for transformation into AML and hence increases genomic instability, defined as the tendency of a genome to acquire genetic alterations which means that the cell’s DNA is more prone to errors during replication and cell division. We have demonstrated that S100A9 in MDS also leads to the development of RNA:DNA hybrids [30] and genomic instability, defined by γH2AX activation which is a marker of DNA damage response [31]. This is reduced with TP-6379 treatment of MDS preclinical specimens (Figure 4I). In addition to HSCPs and MDSCs, among the other cells analyzed by flow cytometry after TP-6379 treatment of MDS BM-MNCs, we found a significant upregulation of T cells, including CD4^+^ and CD8^+^ T cells, and NKT CD8^+^ cells. This increased abundance of lymphocytes may be an important marker for the development of anti-tumor responses that prevent further disease development; this should be further studied in the future. rhS100A9 treatment of healthy BM-MNCs did not induce an overall change in T cell populations but there was a slight, yet significant, increase in overall T cells after TP-6379 treatment (Supplementary Figure S3B). However, rhS100A9 did induce an increase in Tregs, as expected of its suppressive function, and this was reduced by TP-6379 treatment (Supplementary Figure S3C).

### 3.5. Spliceosomal Mutation Has Higher Sensitivity to S100A9/TGFβ Signaling and Downstream Activation of the Mobilizing Agent PAI-1

**Figure 5:**
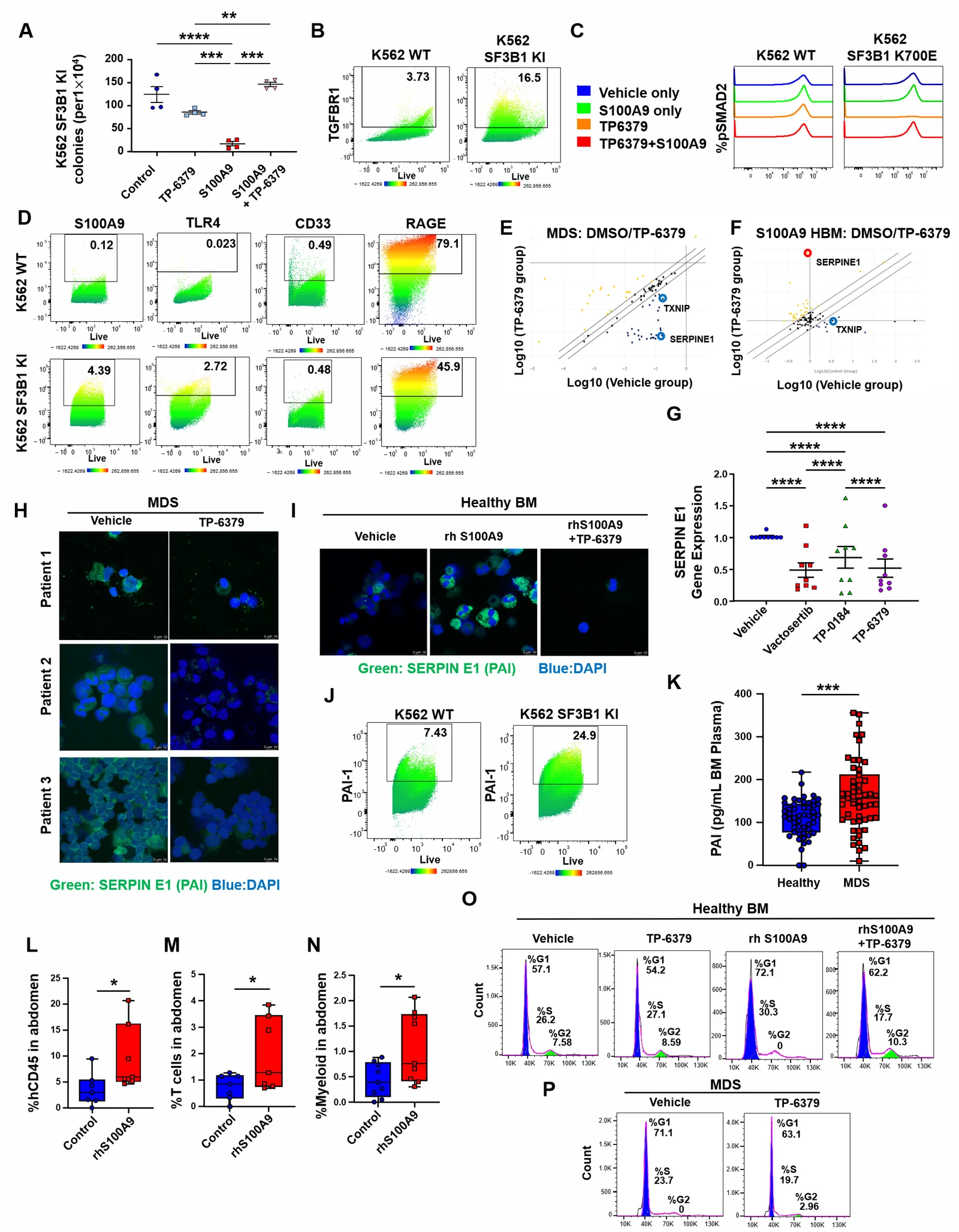
**SF3B1 sensitivity is due to higher TGFBR1 receptor expression and PAI-1 induction.** (**A**) Colony-forming capacity of K562 SF3B1 K700E CRISPR KI mutant cells treated with TP-6379 for 48 h (0.1 μM) only or in the presence of 10 µg/mL of rhS100A9. (**B**) TGFBR1 expression by flow cytometry of K562 WT and SF3B1 CRISPR KI mutant cells (from live gate). (**C**) Flow cytometric analysis of phosphorylated SMAD 2 (pSMAD2) activation in K562 WT and SF3B1 mutant alone or treated with TP-6379 in the presence or absence of rhS100A9. (**D**) Comparative receptor expression of WT (top panels) and SF3B1 mutant K562 cells (bottom panels) for expression of S100A9 and its key receptors TLR4, CD33 and RAGE. (**E**) MDS BM-MNCs were treated in vitro for 48 h with TP-6379 followed by RNA isolation for analysis of TGFβ-related gene expression profile using the Qiagen RT Profiler qPCR array. Results from three separate donors were analyzed in their Geneglobe platform comparing control versus TP-6379-treated cells. (**F**) Same analysis as in (**E**) but with healthy BM-MNCs treated with rhS100A9. (**G**) Validation of array results via qPCR analysis of SERPINE1 expression in primary MDS BM-MNCs treated with either Vactosertib, TP-0184 or TP-6379 (*n* = 10). (**H**) Immunofluorescence staining of three separate MDS BM-MNC specimens treated with 0.1 μM of TP-6379 for 48 h prior to collection and cytospinning onto slides. Cells were then fixed and stained with an anti-PAI-1 primary antibody and an Alexa 488 secondary antibody (green). Nuclei were counterstained with DAPI (blue) and analyzed by confocal microscopy. (**I**) Healthy BM-MNCs were treated with 10 µg/mL of rhS100A9 with or without TP-6379 and analyzed as in (**H**). (**J**) PAI-1 expression analysis of K562 WT and SF3B1 mutant cells by flow cytometry gated on live cells. (**K**) PAI-1 secretion by ELISA of BM plasma from either healthy (*n* = 48) or MDS (*n* = 48) specimens. NSG mice were injected with 1 × 107 PBMC from healthy human donors via tail vein injection. After 24 h 50 µg of recombinant human S100A9 was injected intraperitoneally and after 72 h mice were euthanized and human CD45 (hCD45^+^) cells were measured in the spleen and from abdominal washes. Myeloid cells (CD11b^+^) and T cells (CD3^+^) were also analyzed. Cells are presented as percentages of live cells for (**L**) hCD45^+^ in the abdomen, (**M**) T cells in the abdomen, and (**N**) myeloid cells in the abdomen. (**O**) Healthy BM-MNCs were treated with 10 µg/mL of rhS100A9 with or without TP-6379 for 48 h followed by cell cycle analysis by flow cytometry (representative of *n* = 3). (**P**) MDS BM-MNCs treated with TP-6379 and analyzed for cell cycle changes by flow cytometry. *p*-values are shown as asterisk: * *p* ≤ 0.05, ** *p* ≤ 0.01, *** *p* ≤ 0.001, and **** *p* ≤ 0.0001 as summarized in Graphpad Prism.

Taking advantage of the erythroid progenitor K562 cell line that has an available SF3B1 CRISPR KI mutant counterpart, we tested colony formation after treatment with TP-6379 in the presence or absence of rhS100A9. Both WT and SF3B1 K700E CRISPR KI K562 cells were sensitive to rhS100A9 which reduced colony-forming capacity, although SF3B1 K700E CRISPR KI cells were already reduced compared to WT K562 colonies (compared average of 700 in WT to 200 in SF3B1 K700E CRISPR KI) (Figure 5A, Supplementary Figure S4A). TP-6379 treatment did not restore the colony-forming capacity of WT cells treated with rhS100A9, but it did restore the colony-forming capacity of SF3B1 K700E CRISPR KI cells treated with rhS100A9, akin to our observations with MDS primary specimens. This correlates with higher expression levels of ALK5/TGFBR1 in SF3B1 K700E CRISPR KI cells (Figure 5B), compared to WT K562 cells, which make these cells more sensitive to SMAD2 inactivation by TP-6379 (Figure 5C). The inhibitory effect of TP-6379 can be abrogated by external rhS100A9 addition, establishing its role in the TGFBR1 pathway (Figure 5C). To understand the higher sensitivity of SF3B1 K700E CRISPR KI cells we tested the expression of intracellular S100A9 as well as S100A9-receptors TLR4, CD33 and RAGE. We found that only TLR4 and S100A9 were higher in expression in the SF3B1 K700E CRISPR KI mutant cells, compared to WT K562, while CD33 had no change and RAGE was decreased (Figure 5D). Treatment with S100A9 or TP-6379 did not change the expression of any of these markers (Supplementary Figure S4B).

To understand which components of the TGFβ pathway are relevant in TP-6379 inhibition in low-risk MDS we performed an expression analysis using the TGFβ signaling pathway RT2 Profiler qPCR array (Qiagen, GeneGlobe ID—PARN-235Z) with three MDS specimens treated ex vivo with TP-6379 or vehicle control. We found two genes that were linked to TP-6379 TGFBR1-mediated inhibition: serine protease endothelial plasminogen activator inhibitor E1 (SERPINE1), which codes for plasminogen activator inhibitor-1 (PAI-1) and Thioredoxin-interacting protein (TXNIP) (Figure 5E). These genes are listed as part of the regulation of the proliferation and migration of the TGFβ signaling pathway RT2 Profiler qPCR array. To understand the role of S100A9 we repeated the array with primary healthy BM-MNCs treated with recombinant S100A9. Only SERPINE1, but not TXNIP, was specifically upregulated by S100A9 (Figure 5F). TP-0184 was also able to downregulate SERPINE1 in MDS BM-MNCs treated with TP-0184 and healthy BM-MNCs treated with TP-0184 in vitro in the presence of S100A9 (Supplementary Figure S4C,D). Comparison of untreated bone marrow and TP-0184-treated bone marrow in the presence of S100A9 showed no difference suggesting that they revert to the original expression levels (Supplementary Figure S4E). To validate the reduction in SERPINE1 after treatment we tested low-risk MDS BM-MNCs with Vactosertib, TP-0184 and TP-6379. Each significantly reduced SERPINE1 expression (Figure 5G). The same qPCR expression analysis for TXNIP showed this was not significantly affected by either treatment (Supplementary Figure S4F). SERPINE1 changes matched PAI’s protein expression in MDS BM-MNCs and healthy BM-MNCs treated with rhS100A9 (Figure 5H,I). To confirm that these changes are also linked to the spliceosomal mutations we assessed PAI-1 expression in K562 cells WT or SF3B1 K700E CRISPR KI cells and the mutant was shown to have higher PAI-1 expression compared to WT cells (Figure 5J).

PAI-1 has been demonstrated to play a role in hematopoiesis and mobilization of HSPCs [32] and we wanted to understand if it is also secreted in the bone marrow microenvironment of MDS patients. We measured PAI-1 expression by ELISA with a cohort of healthy donor bone marrow plasma and compared it with PAI-1 levels from a cohort of MDS BM plasma. We observed an increase in the secretion of PAI-1 in the marrow of MDS patients (Figure 5K) consistent with our earlier observations of higher S100A9 [4,5]. To confirm that S100A9 can mobilize inflammatory cells to the microenvironment we performed an in vivo assay where NSG mice were injected with healthy human donor peripheral blood mononuclear cells (PBMCs) intravenously 24 h prior to intraperitoneal injection of rhS100A9. We then assessed the levels of human CD45^+^ cells in both the abdominal cavity and the spleens, where they normally home to after injection, and saw an increase in human CD45^+^ cells in the abdominal cavity when rhS100A9 was present (Figure 5L), with a concomitant decrease in human CD45^+^ cells in the spleens (Supplementary Figure S4G). To test if the recruitment was cell-type-restricted, we measured CD3^+^ as a marker for T cell recruitment (Figure 5M) and CD11b^+^ as a marker for myeloid cell recruitment (Figure 5N) within the human CD45^+^ compartment. Both groups were significantly recruited to the abdominal cavity with a decrease in spleen (Supplementary Figure S4G–I) suggesting overall recruitment of inflammatory cells by S100A9.

PAI-1 has also been shown to be important in cellular senescence through the upregulation of the cell cycle repressors p53 and p21 [33], which have also been linked to S100A9 [34]. Treatment with S100A9 was able to induce cell cycle arrest at G1 in healthy BM-MNCs, which can be overcome by TP-6379 treatment (Figure 5O). Moreover, MDS BM-MNCs which were already high in G1 phase increased in G2 after treatment with TP-6379 (Figure 5P). This demonstrates that the ability of S100A9 to mobilize as well as arrest cell cycle in BM-MNCs can be overcome by ALK5/TGFBR1 inhibition with TP-6379.

## 4. Discussion

In the present study we found that ALK5/TGFBR1 inhibition with TP-6379 is active in improving S100A9-mediated defective hematopoiesis in primary low-risk preclinical MDS specimens. We compared the effectiveness of inhibiting ALK2, ALK4 and ALK5/TGFBR1 in restoring hematopoiesis and found that inhibiting ALK5/TGFBR1 with the investigational compound TP-6379 was less toxic and more efficient at increasing the hematopoiesis potential of primary low-risk MDS BM-MNCs treated ex vivo. Importantly, TP-6379 was also able to restore healthy hematopoiesis abrogated by the presence of rhS100A9. The effectiveness of TGFBR1 inhibition was validated in vivo in the MDS murine model and the S100A9-Tg mice, where there was an improvement of anemia, matched by increased HSPCs and decreased MDSCs in both bone marrow and spleen of the mice. While ALK2 inhibition with TP-0184 was able to reduce MDSCs it did not significantly improve HSPCs and hematopoiesis at the same dose as TP-6379. Therefore, inhibition of TGFBR1 is an important target not only for low-risk MDS but to mediate the effects of TGFβ mediated by excess S100A9 in the microenvironment, through reduction in the immunosuppressive feedback loop characteristic of MDS.

**Figure 6:**
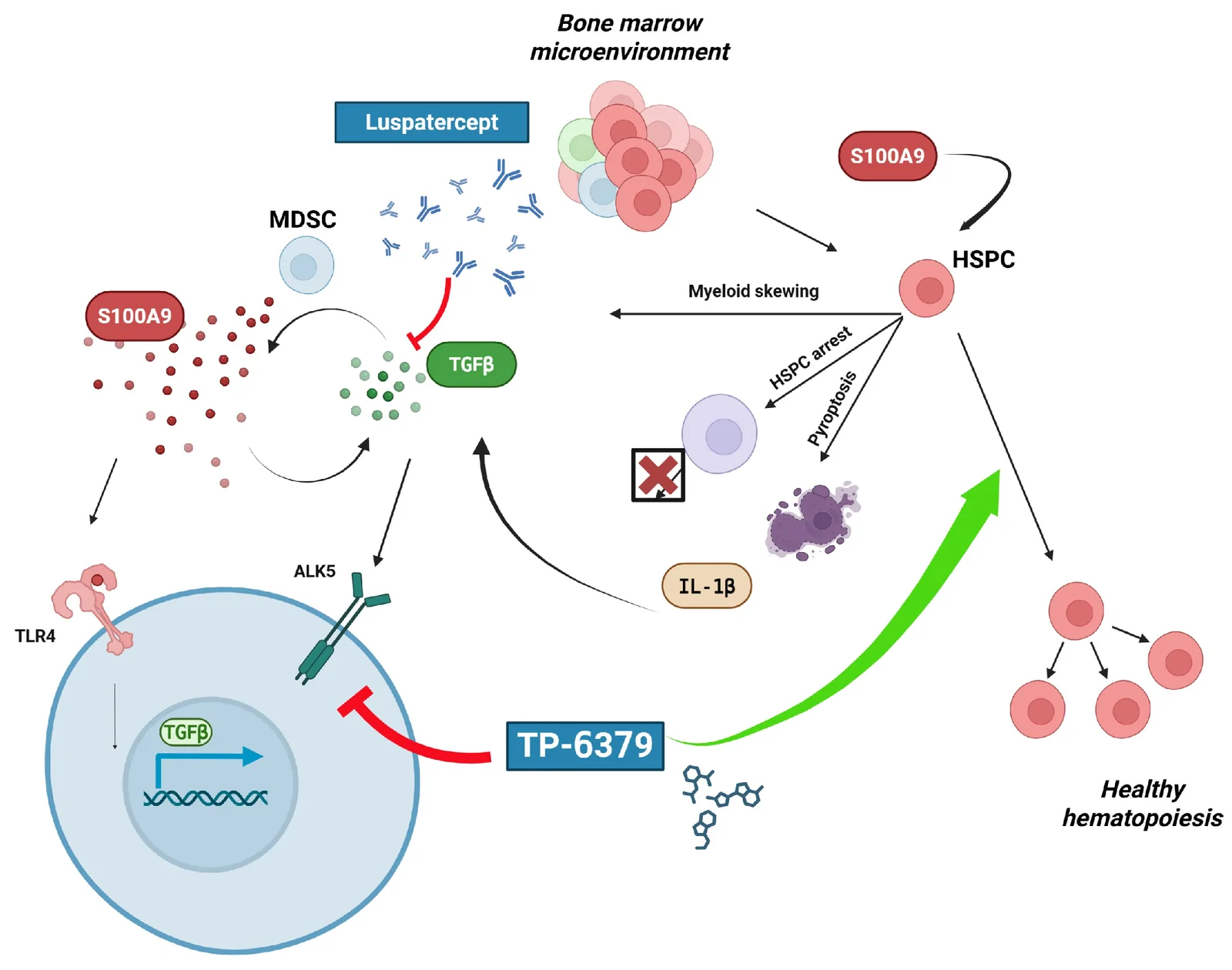
**Schematic representation of findings.** SF3B1 S100A9 mediates cell death and myeloid skewing of HSPCs. This gives rise to accumulation of MDSCs and feedback increases in S100A9 which lead to activation of the TGFβ promoter. Excess TGFβs cooperatively work with S100A9 in the ALK5 receptor to enhance immunosuppressive pathogenesis. While therapies like Luspatercept traps excess TGFβs, TP-6379 goes below the cooperation of S100A9 and TGFβs to reduce their feedback loop aiding in the restoration of healthy hematopoiesis.

As schematized in Figure 6, S100A9 mediates defects in HSPCs, which include pyroptosis, cell cycle arrest and myeloid skewing [4,5,35]. The last one gives rise to increases in immature myeloid cells which include MDSCs which are recruited by and main producers of S100A9 which interacts with TLR4 to induce the expression of TGFβ [4]. This cytokine itself has been shown to synergize with S100A9 through TGFBR1/ALK5 to enhance immunosuppression. Currently used therapies aimed at TGFβ are ligand traps that target the excess of this cytokine only and while effective it has some resistance. Using an ALK5 inhibitor like TP-6374 prevents the feedback loop and cooperation with S100A9 which is likely a cause of its ability to work on Luspatercept-resistant specimens.

Previously others have shown that TGFBR1 inhibition can lead to improved hematopoiesis in low-/int1-risk MDS, which was confirmed by our studies [11]. They also showed that in general MDS specimens have high SMAD2 activation consistent with the role of TGFβ activation in the disease. We expand on that information to show the link with S100A9, which we have shown plays a key role in MDS by inducing death of hematopoietic progenitors directly, through pyroptosis induction and indirectly by MDSCs [2,4,11]. Consistent with the role of SMAD2 activation in MDS [11], S100A9 is also able to induce activation in both HSPCs and MDSCs and can be reduced by TP-6379’s ALK5/TGFBR1 inhibition. This link to S100A9’s immunosuppressive activities may explain why higher risk samples are resistant to ALK5/TGFBR1 inhibition as S100A9’s effects are not as pronounced in higher-risk and transformed disease [5,11,36]. The relevance of ALK5/TGFBR1 inhibition, compared to other ALK family member inhibition, may be due to the fact that most of the TGFβ pathway signaling occurs through ALK5/TGFBR1 [37]. In our testing we found similar effects of each of the inhibitors tested through increased hematopoiesis, reduced immunosuppressive MDSCs and downregulation of PAI-1. However, it was ALK5/TGFBR1 inhibition through TP-6379 that had the most potency of the three inhibitors tested, suggesting that generally targeting the whole TGFβ pathway may reduce the activity of these types of inhibitors, emphasizing the need for ALK5/TGFBR1-specific inhibition.

A case for the role of S100A9-mediated innate immune activity playing a role in the sensitivity of TP-6379 was the observation that MDS BM-MNCs with spliceosomal mutations, including SF3B1, have elevated sensitivity to ALK5/TGFBR1 inhibition. Spliceosomal mutations play a critical role in MDS, and SF3B1 mutations correspond to 35–43% of all MDS cases and almost all of the MDS specimens with ring sideroblasts [8,38,39]. They are known to affect innate immune responses through IRAK4-NFκB activation blocking hematopoietic differentiation [40]. Spliceosomal mutations have also been shown, both in vitro and in vivo models, to activate innate immune responses [41]. Using K562 WT and SF3B1 K700E CRISPR KI cells we demonstrated that this increased sensitivity is likely due to higher ALK5/TGFBR1 expression, as well as increased S100A9 and TLR4, in the spliceosomal mutant cells. In the Luspatercept MEDALIST clinical study the patients with better responses had lower transfusion burden, required less erythropoietin supplementation, had higher chance of SF3B1 mutations and a more recent post diagnosis. This suggests that S100A9-mediated innate immune activity is at its peak sensitizing the cells to the activity of TGFβ inhibition through the upregulation of immunosuppressive mediators [18]. One important observation was the lack of a strong difference between 75 mpk and 150 mpk, which in some cases matches some of the distinctions observed clinically by Luspatercept. This suggests that the lack of a strong dose-dependent response suggests that other factors in the microenvironment may be changing the balance of benefit of TP-6379.

Future studies on this compound should investigate how different cellular components or the levels of factors such as the quantity of circulating S100A9 may affect the response to TP-6379. Reduction in immunosuppression is also evidenced by an increase in lymphocyte populations, such as cytotoxic CD8^+^ T cells and NKT cells, which can be key in preventing the rise in malignant clones. TGFβ signaling is known to affect cellular cytotoxicity including its activation [9]. However, it is important to note that a limitation of our study in this aspect is that we only observed an increase in the abundance of T cells and not their functional state which makes it hard to currently assess their anti-tumor potential. Future studies should focus on understanding the role of ALK5/TGFBR1 inhibition in the functional phenotype of lymphocytes in that context. Another aspect that will need to be addressed in more detail is the potential changes in the myeloid skewing characteristic of MDS. One observation is that the level of BFU-E colonies is increased at the same time compared to CFU-GM colonies and their ratio has been demonstrated to be a potential marker for characterizing myeloid skewing [42]. Importantly, this increases the corelate with a significant decrease in MDSC populations again suggesting that the increase in CFU-GM is due to healthier myelopoiesis, a critical issue with many therapeutic strategies that lead to neutropenias [43]. Further evaluation of specific populations in peripheral blood of MDS specimens would be important. However, our study was limited as that was not collected in our protocol. However, S100A9-Tg mice did show changes in their complete blood counts as well as spleen and bone marrow.

Expression analysis of primary MDS, S100A9-treated healthy BM-MNCs, and K562 SF3B1 K700E mutant cells revealed elevated expression of SERPINE1 coding for PAI-1, which was rescued by TP-6379 treatment. PAI-1 is part of the fibrinolytic system where it has varied physiological roles beyond Fibrin lysis during wound repair [44,45]. As part of the wound repair process PAI-1 has been shown to play key roles in recruitment of HSPCs to the injury site and initiate inflammatory cascades that can aid in the repair process in a TGFβ-mediated manner [45]. For the first time we show that S100A9, which can activate TGFβ and PAI-1, can induce the mobilization of immune cells (lymphoid and myeloid) from the periphery or immune organs (spleen) to the bone marrow confirming our observations of increased recruitment of inflammatory cells to the bone marrow in MDS. This suggests that S100A9 induces an active recruitment of inflammatory cells that occupy the marrow niche and exacerbate the anemic state in MDS pathogenesis. Therefore, ALK5/TGFBR1 inhibition may aid in the release of HSPCs that have been retained in the bone marrow microenvironment in vivo, in addition to its role in improving healthy hematopoiesis. Apart from mobilization, PAI-1 expression downstream of S100A9 likely aids in the HSPC suppression as shown in our study. As shown for TGFβ [11], S100A9 is also linked to cell cycle arrest. PAI-1 can induce cellular senescence as well by preventing the degradation of P53 [46,47] and TGFβ has been shown to directly inhibit HSPC growth through the upregulation of cyclin-dependent kinases maintaining their quiescence [48]. We found that S100A9 can arrest the cell cycle of primary BM-MNCs matching the reduced cycling by MDS BM-MNCs. This quiescent state can be overcome by treatment with TP-6379. Therefore, S100A9 through downstream activation of TGFβ can induce the senescent state of HSPCs suggesting the bone marrow microenvironment has a high degree of immunosuppressive cells recruited by this molecule while at the same time inducing the senescence of HSPCs in the microenvironment.

## Data Availability

Data is contained within the article and Supplementary Materials. If further information is needed the corresponding authors will be ready to provide further information. Except for primary specimens, all the materials used are commercially available as materials and methods. All data and materials used in the analysis will be available to researchers upon request under a material transfer agreement through Moffitt Cancer Center for noncommercial purposes.

## Supplementary Materials

The following supporting information can be downloaded at https://www.mdpi.com/article/10.3390/cells15181674/s1, Figure S1: Treatment of cells with TP-6379 shown to increase hematopoietic colony-forming capacity in primary MDS explants; Figure S2: TP-6379 shown to improve hematopoiesis in S100A9-Tg mice; Figure S3: MDSCs decrease and lymphocytes increase after TP-6379 treatment of primary BM-MNCs; Figure S4: SF3B1 sensitivity is due to higher TGFBR1 receptor expression and PAI-1 induction; Table S1: Specimens used in colony assay; Table S2: Specimens used in flow cytometry analysis.

## Abbreviations

The following abbreviations are used in this manuscript:

MDS	Myelodysplastic syndrome
HSPC	Hematopoietic stem/progenitor cells
BM-MNC	Bone marrow mononuclear cells
rh	Recombinant human
TGFβ	Tumor growth factor β
S100A9-Tg	S100A9-transgenic
KI	Knock-in
TGFBR1	Tumor growth factor β receptor 1
MDSC	Myeloid-derived suppressor cells
IL-1β	Interleukin-1β
ESA	Erythroid-stimulating agents
IRB	Institutional Review Board
MCC	Moffitt Cancer Center
SEM	Standard error of the mean
ANOVA	Analysis of variance
PBS	Phosphate-buffered saline
DMSO	Dimethyl sulfoxide
